# How actions shape perception: learning action-outcome relations and predicting sensory outcomes promote audio-visual temporal binding

**DOI:** 10.1038/srep39086

**Published:** 2016-12-16

**Authors:** Andrea Desantis, Patrick Haggard

**Affiliations:** 1 Institute of cognitive neuroscience, University College London, London, UK

## Abstract

To maintain a temporally-unified representation of audio and visual features of objects in our environment, the brain recalibrates audio-visual simultaneity. This process allows adjustment for both differences in time of transmission and time for processing of audio and visual signals. In four experiments, we show that the cognitive processes for controlling instrumental actions also have strong influence on audio-visual recalibration. Participants learned that right and left hand button-presses each produced a specific audio-visual stimulus. Following one action the audio preceded the visual stimulus, while for the other action audio lagged vision. In a subsequent test phase, left and right button-press generated either the same audio-visual stimulus as learned initially, or the pair associated with the other action. We observed recalibration of simultaneity only for previously-learned audio-visual outcomes. Thus, learning an action-outcome relation promotes temporal grouping of the audio and visual events within the outcome pair, contributing to the creation of a temporally unified multisensory object. This suggests that learning action-outcome relations and the prediction of perceptual outcomes can provide an integrative temporal structure for our experiences of external events.

Our perceptual world is composed of objects consisting of visual, auditory, and other features. These perceptual features are partly processed in separate regions of the brain, yet we experience coherent, multisensory objects. This suggests that, when two sensory inputs are presented, the brain must assign them either to the same multimodal object or to two different unimodal objects.

Multisensory grouping strongly depends on temporal co-occurrence^1^,^2^. However, different sensory modalities, such as visual and auditory inputs, are processed at different latencies, due to differences in the transmission time of light and sound energy, and in peripheral transduction delays^3^,^4^. Such asynchronies represent a challenge for multisensory object perception. However, the brain can meet this challenge by recalibrating audio-visual simultaneity^5^. Notably, after a repeated exposure to a fixed time lag separating an audio and a visual stimulus, the brain adjusts for the lag, so that the stimuli are perceived as more synchronous, compared to initial exposure^5^,^6^,^7^. For instance, repeated exposure to stimuli in which audio precedes vision causes subsequent test stimuli with similar temporal patterns to be perceived as simultaneous, showing that the brain recalibrated audio-visual simultaneity (Fig. 1). Thus, the brain (temporally) integrates audio and visual inputs that are considered to belong to a single physical source object^5^. Several other factors also influence audio-visual temporal recalibration, and these can also be understood under the general concept of attribution of signals to sources. For instance, Heron *et al*.^8^ showed that humans can simultaneously adapt to two opposite timing relationships if these relationship are separated in space.

Here, we investigate another possible driver of binding between sensory signals, namely participants own actions. Humans are not simply passive perceivers but also agents, who bring about sensory events through their own voluntary actions. In our everyday life, our actions mostly generate multiple sensory outcomes (e.g., playing music produces tactile and auditory feedbacks). The human brain can accurately predict what these outcomes will be. We hypothesised that when planning and executing an action the action system might also predict multiple sensory effects (i.e., tactile and auditory outcomes) to occur *together* as a *common* consequence of our motor command. In other words, because we expect that our voluntary actions produce some combination of sensory features (e.g., visual and auditory sensations), the mind may therefore bind these sensory features into a simultaneous multimodal percept. Thus, predicting *what* sensory outcomes our actions generate may have the important consequence of promoting the temporal unity, or simultaneity, of perceptual experience. In this study, we use the term action-outcome prediction to refer to the ability to predict the content of a sensory event caused by one’s own action.

Although, the influence of actions on crossmodal interactions is under-researched, evidence suggests that the action system may influence multimodal processing^9^,^10^,^11^. Indeed, several studies suggest that action processes implicated in the prediction of sensory action-outcomes modulate sensory processing^12^. For instance, predicted unimodal sensory outcomes are attenuated compared to unpredicted and externally generated stimuli^12^,^13^,^14^. Thus, processes involved in the prediction of the specific sensory action-outcome that an action generate, can influence our perceptual experience^15^. These findings have been explained by a widely supported theory of action control according to which during action preparation the action system predicts the specific sensory consequences of an ongoing action^16^,^17^. Predicted outcomes are then compared to the actual outcomes generated by the action allowing for sensorimotor control and learning.

To investigate the role of action processes on multimodal binding, we tested whether the ability to predict the pair of audio-visual stimuli caused by one’s own action would boost temporal integration between the individual stimulus components of the pair, compared to a case in which the audio-visual pair is an unexpected consequence of action. The prediction of sensory outcomes was tested using a (*mis)match design* in which we manipulated the match/mismatch between actual and expected sensory action-outcome^12^. Particularly, in an initial learning phase, participants’ left and right button-presses triggered each a specific audio-visual pair. They learned that one audio-visual pair (e.g., high-pitch tone and yellow flash) was triggered by a left hand action, and the other pair (i.e., low-pitch tone and blue flash) by a right hand action. Importantly, as for the classical temporal recalibration paradigm (Fig. 1), the visual and auditory components of each audio-visual pair were asynchronous: one action caused a sound followed by a flash, while the other action caused a flash followed by a sound. In a subsequent test phase, each button-press produced either the same audio-visual outcome as in the learning phase (“predicted pair”), or the audio-visual pair that was triggered by the other button-press in the learning phase (“unpredicted pair”). It is important to note that the relation between visual and auditory components within each audio-visual pair remained unbroken during the whole task. Notably, during the test phase, it was only the relation between action and audio-visual outcome that was either violated (“unpredicted pair”) or respected (“predicted pair”). Importantly, in the test phase we varied the time interval between the visual and auditory components of each audio-visual pair. Participants were asked to report whether the audio and visual features of the outcome occurred simultaneously or not. We decided to use a simultaneity judgment task instead of a temporal order judgment task for the following reason (for studies showing that temporal judgments and simultaneity judgments do not reflect the same mechanisms see refs 18, 19, 20, 21 and 22). In a temporal order judgment task, repeated exposure to a sound preceding a light in the learning phase might bias participants in responding that the sound occurs before the flash. However, this bias is less likely with our task since simultaneity judgments are orthogonal to our manipulation in the learning phase (i.e., sound first and sound seconds adaptations).

If learning action – audio-visual pair relations promote the binding of the visual and auditory features of the pair, then the brain would adapt to the time lags between the audio and visual stimuli during learning, leading to changes in time perception at test for predicted, but not for unpredicted audio-visual pairs, i.e., the brain would recalibrate audio-visual simultaneity for predicted than for unpredicted audio-visual outcomes (Fig. 1). Our results are consistent with this hypothesis. Learning and predicting what audio-visual outcomes an action generates promote the binding between the individual sensory components of that outcome.

## Materials and Methods

### Participants

Sixteen participants were tested for an allowance of £ 7.5/h (9 women, average age = 24.69 years, *SD* = 5.72 years). The experiment consisted of two sessions conducted in two days (see supplementary material for inclusion criteria). Each session lasted ~80 min. All participants had normal or corrected-to-normal vision and normal hearing, and were naïve as to the hypothesis under investigation. They all gave written informed consent. The experimental protocol was approved by the research ethics committee of University College London. The study adhered to the ethical standards of the Declaration of Helsinki.

### Materials

Visual stimuli were presented on a 15-in. 60 Hz LCD monitor connected to a PC computer. We used Sennheiser HD201 headphones to present audio stimuli. Stimulus presentation was controlled using the psychophysics Toolbox^23^,^24^ for Matlab 8.2.0.

### Procedure

Audio-visual stimuli were created using 2 pure-tones (frequency: 1600 Hz and 400 Hz) and 2 Gaussian blobs (colour: yellow and cyan). Tones had 74 dB SPL sound intensity level. The size of the Gaussian patches was set to *SD* = 0.38°. Gaussian patches were presented with a luminance level of 52.5 cd/m^2^ in a dark grey background (14 cd/m^2^). Viewing distance was ~60 cm. Tones and blobs stimuli were combined to create two audio-visual outcomes. For half of the participants the yellow patch was combined with the high-pitch tone and the blue patch with the low-pitch tone. For the rest of the participants the reversed mapping was used. We choose blue and yellow flashes because we wanted our stimuli to be easily discriminable from each other. We followed the same logic for the sounds. In addition, we wanted to test the influence of action processes on audio-visual binding using arbitrary pairs.

The experiment was composed of 70 blocks (split over two days). Each block consisted of a (short) test phase preceded by a learning phase.

#### Learning phases

The aim of the learning phase was to led participants learn action-audio-visual pair associations. In each trial a rhomb (width: 0.65°, thickness: 0.06°) was presented at the centre of the screen. We used this stimulus as fixation. Participants were asked to execute left or right index finger key-presses. They were instructed that they could choose the action to perform and when to perform it. However, they were told that they had to execute a roughly similar number of left and right key-presses. To help them to achieving this task, we provided a feedback every 10 trials indicating the proportion of right and left button-presses. Inter-trial interval was set to 500 ms.

Depending on the action-outcome mapping subjects were assigned to, each right and left button-press triggered one of the two audio-visual pairs. For example, one group of subjects generated a yellow flash combined with a 1600 Hz tone when executing a left button-press, and a cyan flash combined with a 400 Hz tone when executing a right key-press. The sound and the patch were presented for a duration of ~16 ms. We counterbalanced across participants action - audio-visual outcome mappings.

The visual and audio features of the audio-visual outcomes were separated by a fixed time lag of ~234 ms (this time lag was used based on one of the first study showing audio-visual recalibration^5^). The order of presentation of the auditory and visual components varied as a function of the action that participants executed. For half of the participants, for the left hand action the sound occurred prior to the flash (sound first). Instead, for the right hand action the flash occurred prior to the sound (sound second). For the other half of the participants the reversed action-temporal order mapping was used. The time interval between the action and the presentation of the first stimulus was set to 200 ms (Fig. 2).

The task included 20% of catch trials, to make sure that subjects were paying attention to the audio-visual pair presented. In catch trials a brighter flash or a louder tone was presented (13.3% brighter or louder compared to the standard flashes and sounds presented). Subjects reported the change in stimulus energy by pressing together the right and the left button (Fig. 2). Each learning phase consisted of 20 trials, except for the first block in which subjects completed 40 learning trials.

#### Test phase

Each learning phase was followed by a short test phase, which assessed how the learning of action and audio-visual outcome relations influenced time perception of the visual and audio features of the outcome (Fig. 3). The test phase consisted of 12 trials in the first block, 10 trials thereafter. As for the learning phase audio-visual pairs were presented after participants’ left/right hand actions. Crucially, however, the timing of the auditory stimulus relative to the visual stimulus was randomly varied: −233, −166, −133, −100, −66, −33, 0, 33, 66, 100, 133, 166, or 233 ms. These 13 SOAs were presented 32, 40, 48, 56, 64, 72, 80, 72, 64, 56, 48, 40, and 32 times respectively over the whole experiment. Negative values indicate that the auditory stimulus was presented before the visual stimulus. Subjects reported by pressing one of two foot pedals whether the visual and audio inputs were simultaneous or not (left and right pedal, respectively).

Participants completed “predicted” and “unpredicted” outcome trials in which the associations learned in previous learning phases between left/right action and the subsequent audio-visual pair was respected or violated, respectively. For instance, if in the learning phase the left hand action generated a 1600 Hz tone combined with a yellow patch, then during the test phase the left hand action generated the same yellow flash and 1600 Hz tone on half the trials (“predicted outcomes”), while in the rest of the trials that action was followed by the audio-visual outcome that had previously been associated with the right button-press in the learning phase (“unpredicted outcomes”), i.e., 400 Hz tone and cyan flash (Fig. 3). Importantly, note that the association between visual and auditory components within each pair remained unbroken during the task. In the test phase, we only manipulated the association between button-press and subsequent audio-visual outcome. Notably, this association was either violated (‘unpredicted’ outcome trials) or respected (‘predicted’ outcome trials).

Importantly, subjects were indicated that stimulus identity was irrelevant for the time discrimination task. Nevertheless, they were told to pay attention to stimulus identity because in 20% of the trials, they were asked to report which visual and audio input they generated. We included these catch trials to ensure that subjects paid attention to both predicted and unpredicted audio-visual outcomes.

The sampling of the 13 different audio-visual SOAs (see above) gave a total of 704 trials (i.e., 176 × 2 Adaptation order (sound first, sound second) × 2 Audio-visual pair (predicted and unpredicted)).

## Data Analysis

Participants were tested in a 2 × 2 factorial design with Adaptation order (stimulus order in the learning phase: sound first, sound second) and Audio-visual outcome (predicted, unpredicted) as factors. The proportion of “sound and flash simultaneous” responses was calculated separately for each participant, condition, and audio-visual SOA. Psychometric functions were fitted using a Gaussian model (see formula below) implementing a Least Square Method. Three free parameters were fitted: (1) mean α, (2) standard deviation σ and (3) a scale factor *s*, which refers to the amplitude of the Gaussian curve.


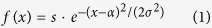


The thirteen audio-visual SOAs we used, were presented 8, 10, 12, 14, 16, 18, 20, 18, 16, 14, 12, 10, and 8 times per condition, respectively. Thus, to minimize the influence of errors on extreme SOAs, each *square error* was weighted according to the number of times that SOA was repeated. Fitting routines were performed using the Matlab functions *fitnlm* and *fmincon*. Mean *r*^2^ for each fit, as a measure of the goodness-of-fit, are reported as follow: predicted outcome sound first condition, *M* = 0.74, *SD* = 0.14; unpredicted outcome sound first condition, *M* = 0.69, *SD* = 0.21; predicted outcome sound second condition, *M* = 0.70, *SD* = 0.20; unpredicted outcome sound second condition, *M* = 0.72, *SD* = 0.15.

Based on each individual function, we calculated the point of subjective simultaneity (PSS) and the temporal sensitivity to audio-visual asynchronies estimated by the Standard Deviation (SD) of the Gaussian fit. Higher SD values in one condition would indicate worse temporal sensitivity in that condition. The PSS was our measure of interest and it corresponds to the mean of the Gaussian distribution. PSS values indicates when the audio input has to be presented with respect to the visual input in order to perceive audio-visual simultaneity. One participant was excluded from the analyses due to poor temporal sensitivity to audio-visual asynchrony (see supplementary material).

## Results

We conducted a repeated measure ANOVA on PSS values with Adaptation order (sound first, sound second) and Audio-visual outcome (predicted, unpredicted) as factors. We observed a significant interaction F(1,14) = 8.697, p = 0.011, 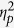 = 0.383, no main effect of Adaptation order and Audio-visual outcome, F(1, 14) = 0.814, p = 0.382, and F(1, 14) = 0.005, p = 0.945, respectively. Paired two-tailed t-tests showed that PSS values shifted toward the adapted lag only when participants were presented with predicted Audio-visual outcomes, t(14) = 3.043, p = 0.009, *d* = 0.452. Notably, subjective audio-visual simultaneity for predicted pairs, when participants were adapted to audio before vision, was 23 ms (on average; positive values indicate sound after the flash). Instead, subjective audio-visual simultaneity for predicted Audio-visual outcomes, when participants were adapted to audio after vision, was 45 ms (Fig. 4). Thus, the adaptation effect for predicted audio-visual outcomes, estimated by the difference between sound first and sound second adaptations, was 22 ms. Importantly, no change in PSS was observed for unpredicted trials (sound first average PSS = 38 ms, sound second average PSS = 29 ms) t(14) = 0.892, p = 0.387. Thus, in our experiment audio-visual recalibration is not based on the stimuli themselves, but it is conditional on the action executed.

We also observed a general bias in all conditions in perceiving audio-visual simultaneity when sounds were presented after the flash (sound first predicted pair: t(14) = 2.132, p = 0.051, d = 0.551; sound first unpredicted pair: t(14) = 2.425, p = 0.029, d = 0.626; sound second predicted pair t(14) = 3.225, p = 0.006, d = 0.833; sound second unpredicted pair t(14) = 2.171, p = 0.048, d = 0.561). This might suggest that participants perceived in general sounds faster than flashes^25^,^26^.

Further analyses of temporal sensitivity and of catch trial performance are reported in the supplementary materials. We also report Bayesian analyses to further explore our effect on PSS values for predicted Audio-visual outcomes.

## Preliminary Discussion

Audio-visual recalibration of simultaneity occurred only when actions triggered the presentation of predicted audio-visual pairs but not when subjects’ actions were followed by unpredicted audio-visual pairs, i.e. the audio-visual outcome associated with the other button-press in the learning phase (see supplementary material for a replication of this finding). This suggests that learning the relation between an action and a specific audio-visual outcome pair drives temporal binding of the visual and auditory inputs *within* the audio-visual outcome. This finding indicates that multisensory temporal binding between vision and audition partly depends on whether or not visual and audio outcomes were predicted by participants’ actions. During the learning phase of the experiment, participants come to learn that a specific audio stimulus and a specific visual stimulus both occur as a *common outcome* of their specific action. We suggest that, as a result of this learning, the perceived temporal delay between the visual and auditory features of the associated audio-visual pair would reduce. Consequently, they would become temporally bound. As a consequence, in the test phase, subjective audio-visual simultaneity would shift toward the adapted lag only for the predicted audio-visual pairs. Indeed, participants perceived more often the audio and visual stimuli as simultaneous when the test phase involved the same audio-visual pair, and a temporal configuration consistent with the audio-visual temporal configuration acquired during the learning phase. Thus, for instance, if audio occurred after vision in the learning phase, then participants would perceive audio-visual simultaneity in the test phase when audio occurred after vision.

However, no difference in PSS was observed for the unpredicted audio-visual pairs. This suggests that recalibration of audio-visual simultaneity is conditional on executing the action that participants had previously learned would produce the audio-visual pair, and was not dependent on the audio-visual pair itself. This corroborates recent studies showing that the identity of the audio-visual pair does not drive temporal recalibration^8^,^27^ (but see ref. 25). In sum, the results suggest that actions specifically bind sensory modalities that occur as their expected consequences.

## Control Experiments

We ran two control experiments to assess whether the audio-visual recalibration of simultaneity we observed was due to action control processes involved in the prediction of sensory outcome rather than based on statistical regularities between a cue (i.e., left/right action) and a subsequent event (audio-visual pair).

## Experiment 2

### Method

In this experiment, participants responded to a visual cue, and caused an audio-visual pair as before, but the identity of the audio-visual pair now depended on the initial visual cue, and not on the action. At the start of the trial, subjects were presented with a hollow circle or a hollow square (0.65° visual angle in diameter, 0.06° line thickness). Visual cues were used as fixation and were randomly presented. After presentation of the visual cue, participants were required to execute a right index finger key-press at a time of their own choosing. Their right key-press produced the occurrence of one of two possible audio-visual pairs. Audio-visual stimuli were contingent with the fixation cues and not with the actions. Notably, each audio-visual stimulus was preceded by a specific visual cue. For example, for some subjects the audio-visual stimulus composed of a 1600 Hz tone and a yellow patch, was preceded by a circle. Instead, the square-cue was associated with a 400 Hz tone paired with a cyan patch. We counterbalanced cue – audio-visual stimulus mappings across participants. Visual cues were presented before action execution to match Experiment 2 with Experiment 1. Recent studies showed that the brain represents predicted outcomes already during motor preparatory processes (~200–250 ms before action execution)^28^,^29^. Thus, we wanted participants to have enough time to process visual cues. Secondly, as in the main experiment audio-visual pairs were generated by an action. This was done to match the attentional demands in the main and the control experiment^12^.

In the test phase, as in the main experiment, subjects completed “unpredicted” and “predicted” audio-visual pair trials, where the previously learned relation between visual cues and audio-visual stimulus was violated or respected, respectively.

Seventeen volunteers were tested (5 males, average age = 21.93 years, *SD* = 3.50 years) for an allowance of £ 7.5/h. All had no hearing impairments and normal or corrected-to-normal vision. They were naïve as to the hypothesis motivating the current research. They all gave written informed consent. The required sample size to achieve a power ≥0.8 was determined using the effect size of the interaction (Adaptation order * Audio-visual pair) observed in the main experiment. The analysis indicated that by testing 16 participants we would have achieved a power of 0.8123. One participant was excluded from the analyses due to very poor temporal sensitivity to audio-visual asynchrony (see supplementary material).

Data was analysed in the same way as in the main experiment. Mean *r*^2^ for each fit was used to assess goodness-of-fit. The values were as follows: sound first predicted stimulus condition, *M* = 0.750, *SD* = 0.172; sound first unpredicted stimulus condition, *M* = 0.753, *SD* = 0.170; sound second predicted stimulus condition, *M* = 0.712, *SD* = 0.200; sound second unpredicted stimulus condition, *M* = 0.790, *SD* = 0.119.

### Results

We conducted a repeated measure ANOVA on PSS values with Adaptation order (sound first, sound second) and Audio-visual pairs (predicted, unpredicted) as factors. We observed no interaction F(1, 15) = 0.003, p = 0.954, no main effect of Adaptation order F(1, 15) = 0.843, p = 0.373, and no main effect of Audio-visual pairs F(1, 15) = 2.441, p = 0.139 (Fig. 5). PSS values were as follows: sound first predicted outcome *M* = 50 ms, *SD* = 44 ms; sound first unpredicted outcome, *M* = 59 ms, *SD* = 47 ms; sound second predicted outcome, *M* = 56 ms, *SD* = 48 ms; sound second unpredicted outcome *M* = 65 ms, *SD* = 54 ms. As for the main experiment, we observed a general bias across all conditions in perceiving audio-visual simultaneity when sounds were presented after the flashes: t(15) = 5.197, p < 0.000, d = 1.299.

Further analyses of temporal sensitivity and of catch trial performance are reported in the supplementary materials. We also report Bayesian analyses to further explore our null effect on PSS values for predicted audio-visual pairs.

### Preliminary discussion

In Experiment 2 no difference in PSS values between sound first and sound second adaptations for the predicted audio-visual pairs was observed. This indicates that visual cues and the simple statistical regularities between a cue and a subsequent audio-visual pairs do not drive temporal binding of audio-visual components.

One possible criticism of Experiment 2 is that we did not entirely match it with the main experiment since visual cues in the control study were presented at the centre of the screen, while the manual actions in our main experiment were clearly lateralized. Recent studies suggest that spatial information might help audio-visual temporal binding^7^,^8^ (but see refs 25 and 30). Thus, we ran a second control study. Now, tactile stimulation of the left or right index finger triggered a bimanual action, which in turn triggered an audio-visual pair. Each specific tactile cue predicted a specific audio-visual pair, but the action had no discriminative association with either the tactile cue or the audio-visual pair.

## Experiment 3

In Experiment 3, the two audio-visual pairs were differentially associated with two tactile stimulations. Tactile stimulations consisted of a 100 ms stimulus with supra-threshold intensity delivered to the dorsal part of the middle phalanx of the left or the right index finger by one solenoid tapper (M&E Solve, UK). In the learning phase, tactile stimulation was presented 350 ms after the onset of the trial (trial onset was indicated with a rhomb presented at the centre of the screen as fixation, see main experiment). The location (left or right) of the tactile stimulation was random, with an equal number of left and right tactile stimulations. Participants were instructed to press both the left and the right keys together at a time of their own choosing, but after the delivery of the tactile stimulus. Their double key-press produced the occurrence of an audio-visual pair. However, audio-visual pairs were now contingent to the tactile stimulus and not to participants’ action. Notably, left and right tactile cues were each followed by a specific audio-visual stimulus. For example, for some subjects the 1600 Hz pure tone combined with a yellow patch was preceded by a left tactile stimulation, and the right-cue was associated with a 400 Hz tone paired with a cyan patch.

Similarly, in the test phase, the location (left/right) of the tactile stimulation was random, with an equal number of left and right tactile stimulations. Participants were instructed to press both the left and the right keys together at a time of their own choosing, but after the tactile stimulation. Their double key-press produced the occurrence of an audio-visual pair. Participants completed “predicted” and “unpredicted” audio-visual pair trials in which the associations learned in previous learning phases between tactile cues and the subsequent audio-visual pair was respected or violated, respectively.

Sixteen volunteers were tested (8 women, average age = 22.5 years, *SD* = 4.59 years) for an allowance of £ 7.5/h. All had normal or corrected-to-normal vision and hearing and were naïve as to the hypothesis under investigation. They all gave written informed consent. One participant was excluded from the analyses due to very poor temporal sensitivity to audio-visual asynchrony (see supplementary material).

Data were analysed in the same way as in the main experiment. Mean *r*^2^ for each fit, as a measure of the goodness-of-fit, are reported as follow: sound first predicted stimulus condition, *M* = 0.771 (*SD* = 0.129); sound first unpredicted stimulus condition, *M* = 0.875 (*SD* = 0.080); sound second predicted stimulus condition, *M* = 0.783 (*SD* = 0.147); sound second unpredicted stimulus condition, *M* = 0.812 (*SD* = 0.135).

### Results

We conducted a repeated measure ANOVA on PSS with two factors: Adaptation order (sound first, sound second) and Audio-visual pairs (predicted, unpredicted). The analysis showed no interaction F(1, 14) = 2.449, p = 0.140 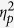 = 0.149 (Fig. 5). Similarly, we observed no main effect of Adaptation order F(1, 14) = 1.013, p = 0.331 and no main effect of Audio-visual pairs F(1, 14) = 0.001, p = 0.977. PSS values observed were as follow: sound first predicted outcome *M* = 55 ms, *SD* = 41 ms; sound first unpredicted outcome, *M* = 64 ms, *SD* = 47 ms; sound second predicted outcome, *M* = 72 ms, *SD* = 45 ms; sound second unpredicted outcome *M* = 63 ms, *SD* = 44 ms. As for the two previous experiments, we observed a general bias across all conditions in perceiving audio-visual simultaneity when sounds were presented after the flash: t(14) = 6.284, p < 0.000, d = 1.623.

Further analyses of temporal sensitivity and of catch trial performance are reported in the supplementary materials. We also report Bayesian analyses to further explore our null effect on PSS values for predicted audio-visual pairs.

## General Discussion

In four experiments, we studied whether learning the association between an action (i.e., left/right button-press) and an audio-visual outcome promote the temporal binding of the visual and auditory features composing the audio-visual outcome. In the learning phase, participants associated a left hand action with a specific pair of visual and auditory stimuli, and a right hand action another specific audio-visual combination. After one action the sound preceded the visual stimulus, while after the other action the visual stimulus preceded the sound. In the subsequent test phase, we varied the temporal interval between the visual and auditory features of these multimodal outcomes. Subjects had to report whether these visual and audio inputs were presented simultaneously or not. In the test phase, participants’ actions could generate the same audio-visual outcome that had previously been associated with that action during the learning phase (“predicted outcomes”), or the audio-visual outcome-pair associated with the other button-press (“unpredicted outcomes”). We measured the point of subjective simultaneity for the audio and visual components in each case.

**Figure 1:**
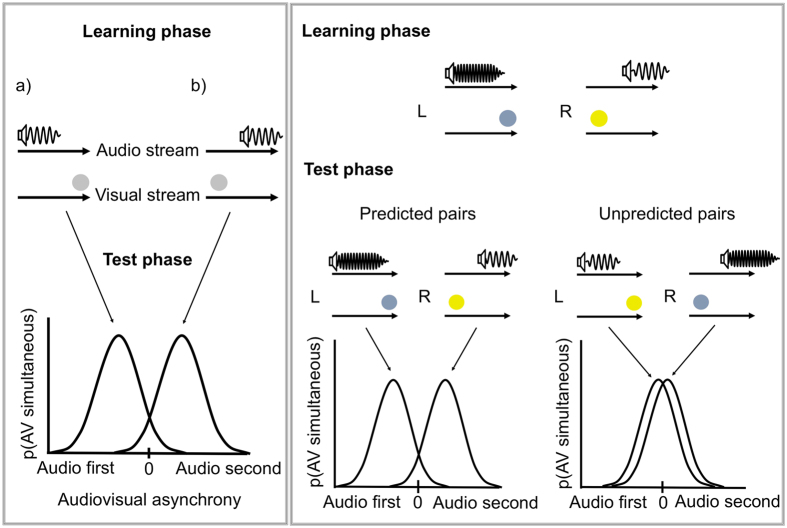
(Left panel) illustration of recalibration of audio-visual simultaneity. In a learning phase audio and visual stimuli are separated by a fixed time lag. In one case, audio leads vision (**a**), in another vision leads audio (**b**). In a test phase audio and visual stimuli are presented at different time intervals and participants are asked to indicate whether or not they are simultaneous. These stimuli are more likely to be perceived as simultaneous (mean of the Gaussian distributions) when presented with a similar temporal configuration to that presented during the learning phase. For instance, when in the learning phase participants are exposed to a sound preceding a light, they will then perceive simultaneity when the sound is presented before the light in the test phase. When exposed to a sound after a light, participants will then perceive simultaneity when the sound is presented after the light. This indicates that the brain reduces the time lag between the two inputs, binding them together in time. (Right panel) schematic of the experimental design. L and R stands for left and right key-presses respectively. Participants learn that a left and a right action each trigger a specific audio-visual pair. In the test phase, we expect recalibration of audio-visual simultaneity when participants’ actions trigger the audio-visual predicted from the instrumental relation learned in the learning phase, but not for the other, unpredicted pair. Learning action-outcome relations and the prediction of sensory outcomes should boost temporal integration of the visual and auditory components of the action-outcome that the action system expects to generate. Stronger recalibration of audio-visual simultaneity should therefore be observed in the test phase for the predicted outcomes.

**Figure 2:**
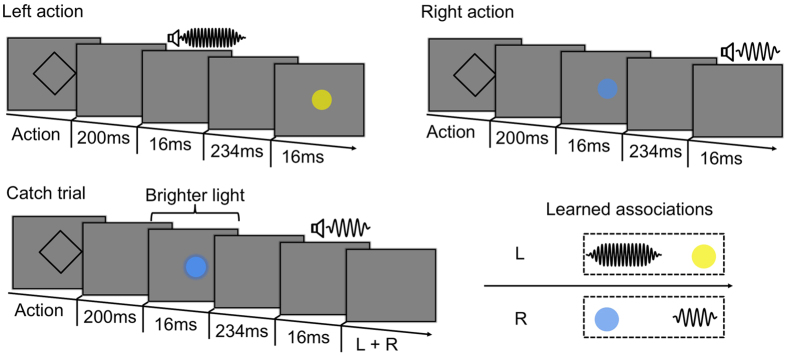
Learning phase. R and L stands for right and left button-presses respectively. Participants were asked to execute left or right index finger key-presses. Left and right actions were associated with specific audio-visual outcomes. For example, one group of subjects generated a yellow flash combined with a 1600 Hz tone when executing a left button-press, and a cyan flash combined with a 400 Hz tone when executing a right key-press. Thus, the action choice determined stimulus identity and order within the outcome pair. The visual and audio components of the pair were separated by a time lag of 234 ms. (Bottom panel). In 20% of the trials (catch trials) participants were presented with either a brighter flash or a louder sound than other trials. They were required to report this increased stimulus energy by executing a simultaneous key-press of both the right and the left button.

**Figure 3:**
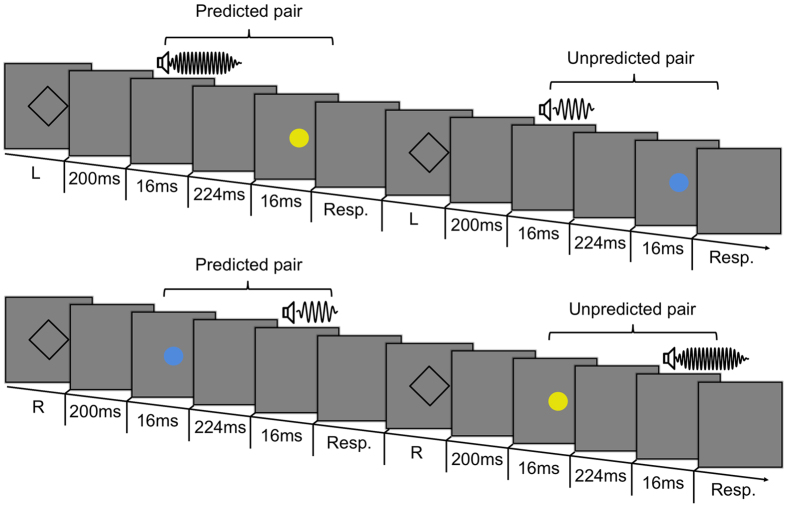
Example of two test trials for left and right hand button-presses. L and R stands for left and right key-presses respectively. In the test phase, participants made left (top panel) or right (bottom panel) hand actions. These were followed either by the predicted audio-visual pair (i.e., the outcome-pair associated with that button-press in the learning phase), or by the unpredicted outcome-pair (i.e., the pair associated with the other button-press in the learning phase). The sound-flash Stimulus Onset Asynchrony (SOA) was varied, to test participants’ perception of audio-visual synchrony.

**Figure 4:**
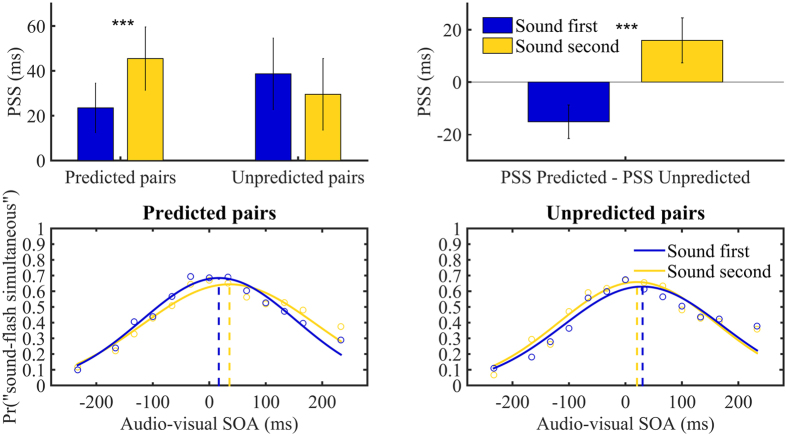
(Top left panel) Mean PSS values for both Adaptation order and Audio-visual outcomes (averaged across all participants). Positive PSS indicates that participants perceived audio-visual simultaneity when sounds were presented after the flash. Bars represents standard errors. (Top right panel) We subtracted PSS values for unpredicted pairs from PSS value for predicted pairs both for sound first and sound second adaptation. Bottom panels depict the proportion of “sound and flash simultaneous” judgments (averaged across participants) for unpredicted pairs (right panel) and predicted (left panel) for both adaptation orders as a function of SOA.

**Figure 5:**
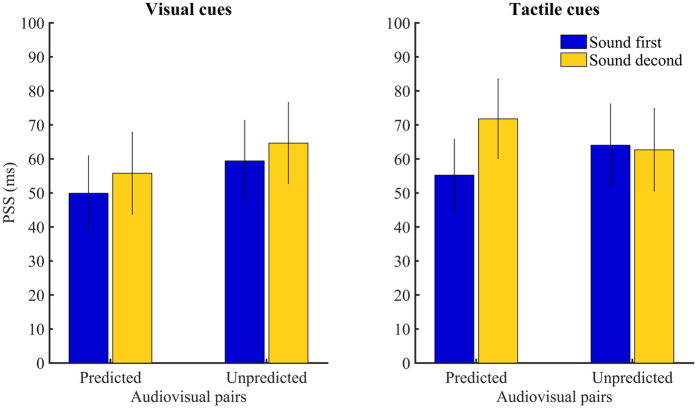
Mean PSS values for both Adaptation order and Audio-visual pairs (averaged across all participants) for Experiment 2 (left panel) and Experiment 3 (right panel). Positive PSS indicates that participants perceived audio-visual simultaneity when sounds were presented after the flash. Bars represents standard errors.

Our results in the test phase confirmed the established finding of recalibration of audio-visual simultaneity^5^. In a test phase, audio and visual stimuli were more likely to be perceived as simultaneous when presented with a similar temporal configuration to that presented during the learning phase, i.e., when in the learning phase participants were exposed to a light preceding a sound, they then perceived simultaneity when the sound was presented after the light in the test phase. Thus, based on prior exposure to a certain audio-visual lag the brain recalibrated its point of subjective simultaneity. Importantly, this shift in perceptual simultaneity occurred only when the audio-visual pair presented at test was caused by the same action that had caused it during the learning phase. Conversely, no shift in perceptual simultaneity was found when the audio-visual pair was triggered by an action different from that which had caused it previously. This suggests that learning the relation between an action and a specific audio-visual outcome pair drives audio-visual temporal binding *within* the outcome pair. More specifically, in the learning phase participants would learn that a specific audio and visual stimulus occur as the *common outcome* of a specific action. As a consequence of this learning, the brain reduces the perceived time lag between those audio and visual outcomes, binding the two events together in time. Accordingly, in the later test phase, audio-visual simultaneity would shift toward the adapted lag only for the audio-visual pair associated with the appropriate action (See Fig. 1 for an illustration). Thus, audio-visual temporal binding between two events is boosted by the predictable associations between an instrumental action and those events. Interestingly, the PSS of unpredicted pairs fell exactly in between the PSS of predicted pairs (see Fig. 4, top right panel). Although, the present three studies do not include a ‘baseline’ phase, in which temporal perception for audio-visual pairs is assessed prior to any learning, the opposite PSS shifts for predicted pairs in the direction of the adapted lag, depicted on the right top panel of Fig. 4, clearly show that audio-visual simultaneity was recalibrated only for predicted pairs. Finally, we observed a general bias in all conditions and all experiments in perceiving audio-visual simultaneity when sounds were presented after the flash. This might suggest that participants in general perceived sounds faster than flashes^25^,^26^.

Interestingly, the temporal binding between audio and visual events remains contingent on the link with the specific action that causes them. Action-induced shifts in perceptual simultaneity did not transfer to a situation where the same events were caused by a different action. This suggests that recalibration of audio-visual simultaneity is not determined only by the identity of the stimuli themselves, but also depends on the identity of the action that caused them. This corroborates recent studies showing that the identity of the audio-visual pair does not drive temporal recalibration^8^,^27^ (but see ref. 25).

Thus, learning a specific relation between an action and the identity of a subsequent audio-visual outcome led to the temporal binding of the visual and auditory components of the outcome. Our control experiments found no corresponding shift in PSS values when specific lagged audio-visual stimuli were associated with visual cues or lateralised tactile cues (Experiment 2 and 3, respectively). To match Experiments 2 and 3 with Experiment 1 we decided to present visual/tactile cues before action execution. Recent studies showed that the brain represents predicted outcomes already during motor preparatory processes^28^,^29^, hence, before action execution. Thus, we wanted participants to have enough time to process visual and tactile cues. Secondly, we did not want the experiments 2 and 3 to be passive. Thus, as in Experiment 1, in those experiments audio-visual pairs were generated by an action. This was done to match the attentional demands across experiments^12^. Indeed, Hughes and his colleagues^12^ showed that comparing active and passive conditions differ in terms of action processes but also in terms of processes such as attention or readiness. Indeed, actions are predictable; instead external cues are unpredictable since we do not control their onset. Unpredictable cues may attract participants’ attention, for example by producing an orienting response. This could, in turn, influence processing of subsequent sensory events. In the current study we wanted to reduce the impact of these attentional factors.

The absence of a shift of PSS in our two control experiments suggest that simple statistical regularities between cues and subsequent events cannot explain the shift in PSS we observed in the main experiment. Interestingly, participants in the control studies did make voluntary actions, although, unlike experiment 1 (and experiment 4 in the supplementary material), this action did not determine the outcome. Indeed, in the control experiments, the actions were not reliably associated with a specific audio-visual pair. These results suggest that mere expectation of an outcome pair did not boost audio-visual temporal binding to the same extent as a voluntary action known to generate the outcome pair. That is, the recalibration of perceived simultaneity was due to the capacity to control outcomes through one’s own voluntary action. Thus, we further conclude that mere motor execution is not sufficient to boost temporal binding.

This form of action-based prediction appears to have a special role in constructing perceptions of the external world. Many studies showed that sensory outcomes of actions are perceived as less intense than control events^13^,^14^,^17^,^28^. Our study identifies a second general way in which action-based predictions structure subsequent perception: action and outcome learning promotes the temporal grouping of the distinct features of a multisensory action-effect into a more temporally bound percept. In this sense, action-related processes structure our perceptual experience, into coherent, grouped units. Previous studies observed that multimodal temporal grouping is crucial for guiding and controlling our actions, since it enables the preparation and execution of accurate and rapid responses^31^,^32^,^33^,^34^. The current study seems to reverse the link between crossmodal interactions and action. Notably, it suggests that action processes linked to the prediction of action-outcomes influence the perceptual mechanisms responsible for multimodal binding^9^,^10^,^11^.

We can only speculate about *how* and *why* action selection promotes multisensory grouping in this way. In terms of *how*, several scenarios could account for our results. One might speculate that motor mechanisms underlying action selection and the prediction of action-outcomes mediate audio-visual temporal binding. A recent fMRI study supports this interpretation. Butler, James, and James^35^ observed that audio-visual object recognition performances improved when participants could actively explore audio-visual objects, compared to when participants could only observe another agent interacting with them (passive observation group). In addition, regions classically involved in the preparation of actions and in the comparison of expected and actual action-outcomes (i.e., the Supplementary Motor Area, the Cerebellum and the Cingulate Gyrus^36^,^37^,^38^) were more strongly activated in the active group compared to the passive group, during the presentation of previously learned audio-visual objects. Interestingly, the active group also showed higher activation of brain areas underlying audio-visual binding (e.g., Superior Temporal Sulcus STS).

We speculate that similar mechanisms explain our findings. It has been shown that mechanisms involved in the preparation and execution of actions modulate the sensory regions representing the expected action-effect, even prior stimulus presentation^29^,^39^,^40^,^41^,^42^,^43^. One might speculate that predicting the outcome of actions modulated brain regions responsible for audio-visual binding. Thus, the components of a predicted multimodal outcome would be perceived more often as occurring together in time compared to sensory features composing an unpredicted outcome.

An alternative (not incompatible) explanation would suggest that action processes promote multisensory integration by allowing humans to extract statistical regularities in the environment faster and more efficiently compared to passive perception or to external control (in Experiments 2 and 3 the identity of the audio-visual outcomes was controlled by visual and tactile cues). Accordingly, one might speculate that a similar audio-visual binding observed in Experiment 1 (and Experiment 4, see supplementary material) could also occur with visual or tactile cues but only after more extensive learning.

In terms of *why*, for instrumental actions to be functional, the mind must learn and maintain correct action-outcome associations. This requires correctly assigning events as either outcomes of one’s own, or external events. Action-based multisensory binding could contribute to this self-attribution process. If one component event can be self-attributed, then other events with which it has been grouped will also be self-attributed. That is, action-based multisensory grouping of events tends to concentrate the evidence required for computing attribution.

Action influences perception in several ways^12^,^44^. Our results identify a novel relation between action and perception. Learning to make outcome-based selections between instrumental actions promotes temporal binding within the different components of the outcome itself. Thus, learning instrumental actions may drive the perceptual binding of information from different sensory modalities, that would otherwise merely appear as temporally correlated. Taken together our results suggest that acquiring and maintaining action-outcome relations engage a specific, integrative cognitive mechanism for perceptual processing of external events. This mechanism might play an important role in producing a coherent perceptual experience.

## Additional Information

**How to cite this article**: Desantis, A. and Haggard, P. How actions shape perception: learning action-outcome relations and predicting sensory outcomes promote audio-visual temporal binding. *Sci. Rep.* **6**, 39086; doi: 10.1038/srep39086 (2016).

**Publisher's note:** Springer Nature remains neutral with regard to jurisdictional claims in published maps and institutional affiliations.

## Supplementary Material

Supplementary Material

## References

[b1] Meredith M. A., Nemitz J. W. & Stein B. E. Determinants of multisensory integration in superior colliculus neurons. I. Temporal factors. J. Neurosci. 7, 3215–3229 (1987). 3668625 10.1523/JNEUROSCI.07-10-03215.1987 PMC6569162

[b2] Stein B. E. The New Handbook of Multisensory Processes. (MIT Press, 2012).

[b3] King A. J. Multisensory integration: strategies for synchronization. Curr. Biol. CB 15, R339–341 (2005). 15886092 10.1016/j.cub.2005.04.022

[b4] Spence C. & Squire S. Multisensory Integration: Maintaining the Perception of Synchrony. Curr. Biol. 13, R519–R521 (2003). 12842029 10.1016/s0960-9822(03)00445-7

[b5] Fujisaki W., Shimojo S., Kashino M. & Nishida S. ’ya. Recalibration of audio-visual simultaneity. Nat. Neurosci. 7, 773–778 (2004). 15195098 10.1038/nn1268

[b6] Di Luca M., Machulla T.-K. & Ernst M. O. Recalibration of multisensory simultaneity: cross-modal transfer coincides with a change in perceptual latency. J. Vis. 9, 7.1–16 (2009). 10.1167/9.12.7 20053098

[b7] Roseboom W. & Arnold D. H. Twice upon a time: multiple concurrent temporal recalibrations of audio-visual speech. Psychol. Sci. 22, 872–877 (2011). 21690312 10.1177/0956797611413293

[b8] Heron J., Roach N. W., Hanson J. V. M., McGraw P. V. & Whitaker D. Audio-visual time perception is spatially specific. Exp. Brain Res. 218, 477–485 (2012). 22367399 10.1007/s00221-012-3038-3 PMC3324684

[b9] Desantis A. & Haggard P. Action-outcome learning and prediction shape the window of simultaneity of audio-visual outcomes. Cognition 153, 33–42 (2016). 27131076 10.1016/j.cognition.2016.03.009

[b10] Petrini K., Russell M. & Pollick F. When knowing can replace seeing in audio-visual integration of actions. Cognition 110, 432–439 (2009). 19121519 10.1016/j.cognition.2008.11.015

[b11] Lewkowicz D. J. & Ghazanfar A. A. The emergence of multisensory systems through perceptual narrowing. Trends Cogn. Sci. 13, 470–478 (2009). 19748305 10.1016/j.tics.2009.08.004

[b12] Hughes G., Desantis A. & Waszak F. Mechanisms of intentional binding and sensory attenuation: the role of temporal prediction, temporal control, identity prediction, and motor prediction. Psychol. Bull. 139, 133–151 (2013). 22612280 10.1037/a0028566

[b13] Blakemore S.-J., Wolpert D. & Frith C. Why can’t you tickle yourself? NeuroReport Rapid Commun. Neurosci. Res. 11, R11–R16 (2000). 10.1097/00001756-200008030-00002 10943682

[b14] Cardoso-Leite P., Mamassian P., Schütz-Bosbach S. & Waszak F. A New Look at Sensory Attenuation. Psychol. Sci. 21, 1740–1745 (2010). 21119181 10.1177/0956797610389187

[b15] Wilson M. & Knoblich G. The case for motor involvement in perceiving conspecifics. Psychol. Bull. 131, 460–473 (2005). 15869341 10.1037/0033-2909.131.3.460

[b16] Blakemore S. J., Wolpert D. M. & Frith C. D. Abnormalities in the awareness of action. Trends Cogn. Sci. 6, 237–242 (2002). 12039604 10.1016/s1364-6613(02)01907-1

[b17] Wolpert D. M. Computational approaches to motor control. Trends Cogn. Sci. 1, 209–216 (1997). 21223909 10.1016/S1364-6613(97)01070-X

[b18] Love S. A., Petrini K., Cheng A. & Pollick F. E. A Psychophysical Investigation of Differences between Synchrony and Temporal Order Judgments. PLOS ONE 8, e54798 (2013). 23349971 10.1371/journal.pone.0054798 PMC3549984

[b19] Petrini K., Holt S. & Pollick F. Expertise with multisensory events eliminates the effect of biological motion rotation on audio-visual synchrony perception. Journal of Vision, 10(5):2, 1–14, doi: 10.1167/10.5.2 (2010). 20616132

[b20] Binder M. Neural correlates of audio-visual temporal processing–comparison of temporal order and simultaneity judgments. Neuroscience 300, 432–447 (2015). 25982561 10.1016/j.neuroscience.2015.05.011

[b21] Maier J. X., Di Luca M. & Noppeney U. Audio-visual asynchrony detection in human speech. J. Exp. Psychol. Hum. Percept. Perform 37, 245–256 (2011). 20731507 10.1037/a0019952

[b22] Fujisaki W. & Nishida S. ’ya. Audio-tactile superiority over visuo-tactile and audio-visual combinations in the temporal resolution of synchrony perception. Exp. Brain Res. 198, 245–259 (2009). 19499212 10.1007/s00221-009-1870-x

[b23] Brainard D. H. The Psychophysics Toolbox. Spat. Vis. 10, 433–436 (1997). 9176952

[b24] Pelli D. G. The VideoToolbox software for visual psychophysics: transforming numbers into movies. Spat. Vis. 10, 437–442 (1997). 9176953

[b25] Roseboom W., Kawabe T. & Nishida S. ’ya. Audio-visual temporal recalibration can be constrained by content cues regardless of spatial overlap. Percept. Sci. 4, 189 (2013). 10.3389/fpsyg.2013.00189 PMC3633943 23658549

[b26] Eijk R. L. J. van, Kohlrausch A., Juola J. F. & Par S. van de. Audio-visual synchrony and temporal order judgments: Effects of experimental method and stimulus type. Percept. Psychophys. 70, 955–968 (2008). 18717383 10.3758/pp.70.6.955

[b27] Yamamoto S., Miyazaki M., Iwano T. & Kitazawa S. Bayesian Calibration of Simultaneity in Audio-visual Temporal Order Judgments. PLoS ONE 7, e40379 (2012). 22792297 10.1371/journal.pone.0040379 PMC3392227

[b28] Bays P. M., Wolpert D. M. & Flanagan J. R. Perception of the Consequences of Self-Action Is Temporally Tuned and Event Driven. Curr. Biol. 15, 1125–1128 (2005). 15964278 10.1016/j.cub.2005.05.023

[b29] Desantis A., Roussel C. & Waszak F. The temporal dynamics of the perceptual consequences of action-effect prediction. Cognition 132, 243–250 (2014). 24853627 10.1016/j.cognition.2014.04.010

[b30] Yuan X., Li B., Bi C., Yin H. & Huang X. Audio-visual temporal recalibration: space-based versus context-based. Perception 41, 1218–1233 (2012). 23469702 10.1068/p7243

[b31] Colonius H. & Arndt P. A two-stage model for visual-auditory interaction in saccadic latencies. Percept. Psychophys. 63, 126–147 (2001). 11304009 10.3758/bf03200508

[b32] Colonius H. & Diederich A. Multisensory interaction in saccadic reaction time: a time-window-of-integration model. J. Cogn. Neurosci. 16, 1000–1009 (2004). 15298787 10.1162/0898929041502733

[b33] Frens M. A., Opstal A. J. V. & Willigen R. F. V. D. Spatial and temporal factors determine auditory-visual interactions in human saccadic eye movements. Percept. Psychophys. 57, 802–816 (1995). 7651805 10.3758/bf03206796

[b34] Stein B. E. & Meredith M. A. Multisensory integration. Neural and behavioral solutions for dealing with stimuli from different sensory modalities. Ann. N. Y. Acad. Sci. 608, 51–65–70 (1990). 10.1111/j.1749-6632.1990.tb48891.x 2075959

[b35] Butler A. J., James T. W. & James K. H. Enhanced multisensory integration and motor reactivation after active motor learning of audio-visual associations. J. Cogn. Neurosci. 23, 3515–3528 (2011). 21452947 10.1162/jocn_a_00015

[b36] Blakemore S. J., Frith C. D. & Wolpert D. M. Spatio-Temporal Prediction Modulates the Perception of Self-Produced Stimuli. J. Cogn. Neurosci. 11, 551–559 (1999). 10511643 10.1162/089892999563607

[b37] Haggard P. & Whitford B. Supplementary motor area provides an efferent signal for sensory suppression. Cogn. Brain Res. 19, 52–58 (2004). 10.1016/j.cogbrainres.2003.10.018 14972358

[b38] Nachev P., Kennard C. & Husain M. Functional role of the supplementary and pre-supplementary motor areas. Nat. Rev. Neurosci. 9, 856–869 (2008). 18843271 10.1038/nrn2478

[b39] Stenner M.-P., Bauer M., Heinze H.-J., Haggard P. & Dolan R. J. Parallel processing streams for motor output and sensory prediction during action preparation. J. Neurophysiol. jn.00616.2014, doi: 10.1152/jn.00616.2014 (2014). PMC4359987 25540223

[b40] Roussel C., Hughes G. & Waszak F. A preactivation account of sensory attenuation. Neuropsychologia 51, 922–929 (2013). 23428377 10.1016/j.neuropsychologia.2013.02.005

[b41] Roussel C., Hughes G. & Waszak F. Action prediction modulates both neurophysiological and psychophysical indices of sensory attenuation. Front. Hum. Neurosci. 8, 115 (2014). 24616691 10.3389/fnhum.2014.00115 PMC3937955

[b42] SanMiguel I., Widmann A., Bendixen A., Trujillo-Barreto N. & Schröger E. Hearing Silences: Human Auditory Processing Relies on Preactivation of Sound-Specific Brain Activity Patterns. J. Neurosci. 33, 8633–8639 (2013). 23678108 10.1523/JNEUROSCI.5821-12.2013 PMC6618825

[b43] Stenner M.-P., Bauer M., Haggard P., Heinze H.-J. & Dolan R. Enhanced Alpha-oscillations in Visual Cortex during Anticipation of Self-generated Visual Stimulation. J. Cogn. Neurosci. 26, 2540–2551 (2014). 24800633 10.1162/jocn_a_00658

[b44] O’Regan J. K. & Noë A. A sensorimotor account of vision and visual consciousness. Behav. Brain Sci. 24, 939–973 (2001). 12239892 10.1017/s0140525x01000115

