# Supplementary material for: How actions shape perception: learning action-outcome relations and predicting sensory outcomes promote audio-visual temporal binding
Source: Sci Rep. 2016 Dec 16;6:39086. doi: 10.1038/srep39086 (PMC5159801; doi:10.1038/srep39086)
Supplement: Supplementary Material [file srep39086-s1.doc]

**Supplementary material**

**How actions shape perception: learning action-outcome relations and predicting sensory outcomes promote audio-visual temporal binding**

Andrea Desantis ^1 *^, Patrick Haggard ^1^

^1^Institute of cognitive neuroscience, University College London, London, UK

Corresponding Author: [*aerdna.desantis@gmail.com](mailto:*aerdna.desantis@gmail.com)

**Experiment 1: Action-outcome associations**

**Participants.** Detecting audiovisual temporal asynchrony was very challenging for some participants. Consequently, when participants in the first test session did not meet the performance level required to be included in the sample, they were not contacted to participate in the second session of the experiment. We used two criteria to assess their performance. The first criterion was their temporal sensitivity to audiovisual asynchrony, measured by the Standard Deviation (SD) of the psychometric curve: SD should not exceed the longest audio-visual SOA we used (i.e., 233 ms). The second criterion was the amplitude/height of the psychometric curve, measured by the scale factor. The amplitude should reach at least ~0.5 proportion of judgment sound-flash simultaneous. Based on these criteria, two participants were initially recruited but were not included in the sample. They did not complete the second session of the experiment due to low temporal sensitivity to audiovisual asynchrony in the first session: their mean Standard Deviation calculated across all conditions was higher than the longest audiovisual SOA (i.e., SD > 233 ms). This indicates that the detection of audiovisual asynchrony was particularly hard for them: even with the longest audiovisual SOA, participants could not detect asynchrony. Moreover, another participant that completed both experimental sessions was excluded from our analyses due to poor temporal sensitivity (SD > 233ms).

**Results.** We conducted a repeated measure ANOVA on temporal sensitivity values (standard deviation of the Gaussian fit). The analyses showed no interaction F(1, 14) = .700, p = .418, no main effect of Adaptation order F(1, 14) = 1.815, p = .199, and no main effect of Audiovisual pairs F(1, 14) = 0.646, p = .435. Thus, participants’ temporal sensitivity was not affected by our manipulation.

Two control analyses investigated whether the differences between predicted and unpredicted outcomes could reflect factors other than the previously-learned action-outcome associations. In order to assess whether participants were equally paying attention to audiovisual pairs in the two adaptation order conditions we conducted a repeated measure ANOVA on the proportion of correct detection of high-saliency catch trials for both sound first (*M* = 0.95, *SD* = 0.06) and sound second adaptation (*M* = 0.93, *SD* = 0.07) in the learning phase. The analysis showed no significant effect of Adaptation order F(1, 14) = 3.4, p = .086,
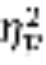
 = 0.195.

Further analyses assessed whether participants paid equal attention to predicted and unpredicted audiovisual pairs in the test phase. A repeated measures ANOVA on identification performances on catch trials with Adaptation order (sound first, sound second) and Audiovisual pairs (predicted, unpredicted) as factors, showed no significant interaction F(1, 14) = .532, p = .477, no main effect of Adaptation order F(1, 14) = 1.687, p = .215, and no main effect of Audiovisual pairs F(1, 15) = 0.69, p = .42. The proportion of correct responses for each condition were as follows: predicted outcome sound first: *M* = 0.96, *SD* = 0.07; unpredicted outcome sound second: *M* = 0.97, *SD* = 0.05; predicted outcome sound first: *M* = 0.97, *SD* = 0.03; unpredicted outcome sound second: *M* = 0.95, *SD* = 0.08.

To further explore our effect on PSS values for predicted audiovisual pairs we conducted Bayesian analyses. We calculated the Bayes factor (Dienes, 2014), to quantify how strongly the data support the temporal recalibration theory or the null hypothesis. To feed our Bayesian analyses we estimated the predictions of the temporal recalibration theory from previous literature that tested audiovisual temporal recalibration using a similar experimental setting as the one we used. From Fujisaki et al., (2004) the predicted mean difference between sound first and sound second adaptations (235ms time lag) was 58ms with a standard error of 28ms. Our analyses showed that the likelihood of the obtained PSS differences between sound first and sound second adaptations (for the predicted pairs), given the expected temporal recalibration difference, was 6.2807. Instead, the likelihood of the obtained data given the null hypothesis was 0.5388. The measured Bayes factor was 11.66. Note that a Bayes factor of more than 3 can be taken as substantial evidence for the theory being assessed, and thus against the null hypothesis. Thus, this analysis strongly suggests that we observe genuinely audiovisual recalibration of simultaneity for predicted pairs.

**Experiment 2: Visual cue-based associations**

**Participants.** One participant was excluded from the analyses due to extremely poor temporal sensitivity (mean SD measured across all conditions), i.e., the SD was higher than the longest audiovisual SOA (233ms). One more participant did not complete the second session of the experiment due to very low temporal discrimination in the first session.

**Results.** We conducted a repeated measure ANOVA on temporal sensitivity values (standard deviation of the Gaussian fit). We observed no significant interaction F(1, 15) = 0.894, p = .359. Similarly, no main effects of Adaptation order F(1, 15) = .004, p = .950 and Audiovisual pairs, F(1, 15) = 0.852, p = .370 were observed.

A repeated measure ANOVA on saliency detection performances for both sound first (*M* = 0.94, *SD* = 0.07) and sound second adaptations (*M* = 0.93, *SD* = 0.08) investigated whether participants equally paid attention to audiovisual pairs in the two adaptation order conditions of the learning phase. The analysis showed no significant effect of Adaptation order F(1, 15) = 1.785, p = .201.

Finally, to assess whether participants were allocating the same amount of attentional resources to predicted/unpredicted pairs in the test phase, we conducted a repeated measure ANOVA on identification performances in the catch trials of the test phase. The analysis showed no significant interaction F(1, 15) = .14, p = .715, no main effect of Adaptation order F(1, 15) = .19 p = .665, no main effect Audiovisual pair F(1, 15) = .10, p = .752. This indicates that participants’ attention was equally focused to stimuli in all conditions. Furthermore, the proportion of correct identification performances showed that stimuli were correctly identified in almost all catch trials (sound first predicted pairs: *M* = 0.97, *SD* = 0.04; sound first unpredicted pairs: *M* = 0.96, *SD* = 0.04; sound second predicted pairs: *M* = 0.97, *SD* = 0.03; sound second unpredicted pairs: *M* = 0.97, *SD* = 0.06).

To further explore our null result on PSS values for predicted outcomes, we calculated the Bayes factor as illustrated in the results of our main experiment. The analyses showed that the likelihood of the obtained PSS for the sound first and sound second adaptations given the expected temporal recalibration effect was 2.7319. Instead, the likelihood of the obtained data given the null hypothesis was 38.0867. Finally, the Bayes factor was 0.07, thus strongly suggesting that mere statistical regularities between visual cues and subsequent audiovisual pairs does not induce a temporal recalibration effects (a Bayes factor of 1/3 or less can be taken as substantial evidence for the null hypothesis.

**Experiment 3: Tactile cue-based associations**

**Participants.** One participant was excluded from our analyses due to poor temporal discrimination performances. Two more participants were recruited but were not included in the sample after the first session.

**Results.** We conducted a repeated measure ANOVA on temporal sensitivity (SD values). The analysis showed no significant interaction F(1, 14) = .384, p = .545. The main effect of Adaption order and Audiovisual pairs were also not significant, F(1, 14) = 1.899, p = .190, and F(1, 14) = 1.789, p = .202, respectively.

A repeated measure ANOVA on catch trial performance for both sound first (*M* = 0.87, *SD* = 0.13) and sound second adaptation (*M* = 0.91, *SD* = 0.07) investigated whether participants were equally able to detect high-salience stimuli, suggesting equal attention to audiovisual pairs in the two adaptation orders in the learning phase. The analysis showed no significant effect of Adaptation order F(1, 14) = 1.191, p = .293.

Finally, to assess whether participants were allocating the same amount of attentional resources to predicted/unpredicted stimuli, we conducted a repeated measure ANOVA on identification performance in the catch trials of the test phase. The analysis showed no significant interaction F(1, 14) = .98, p = .338, no main effect of Adaptation order F(1, 14) = .22 p = .647, no main effect Audiovisual pairs F(1, 14) = 1.99, p = .180. This indicates that participants’ attention was equally focused to stimuli in all conditions. Furthermore, the proportion of correct identification performances shows that stimuli were correctly identified in almost all catch trials (sound first predicted pairs: *M* = 0.96, *SD* = 0.03; sound first unpredicted pairs: *M* = 0.98, *SD* = 0.03; sound second predicted pairs: *M* = 0.97, *SD* = 0.05; sound second unpredicted pairs: *M* = 0.97, *SD* = 0.02).

To further explore our null result on PSS values for predicted pairs we calculated the Bayes factor as illustrated in the results section of our main experiment. The analyses showed that the likelihood of the obtained PSS difference between sound first and sound second conditions, given the expected temporal recalibration difference, was 5.0031. Instead, the likelihood of the obtained data given the null hypothesis was 9.3575. Finally, the Bayes factor was 0.53. This analysis provides modest support for the view that mere statistical regularities between tactile cues and subsequent audiovisual pairs does not induce a temporal recalibration of audiovisual simultaneity.

**Experiment 4**

**Materials and Methods**

**Participants.** The sample consisted of twenty-four participants[[1]](#footnote-2) tested for an allowance of £ 7.5/h (12 women, average age = 21.46 years, *SD* = 2.78 years). Participants completed the experiment in two sessions taking place in two different days. Each session lasted ~90min. All participants had normal or corrected-to-normal vision and hearing and were naïve as to the hypothesis under investigation. They all gave written informed consent. The experimental protocol was approved by the research ethics committee of University College London. The study adhered to the ethical standards of the Declaration of Helsinki.

**Materials.** See Main experiment

**Stimuli and procedure.** We used similar stimuli as in the main experiment, with two important changes. First, we used greater separations between audio and visual stimuli, compared to Experiment 1, with the aim of making simultaneity judgements easier (see later). However, this inevitably reduces the resolution of any individual PSS estimate. Therefore, we collected a larger dataset, compensating for the loss of temporal resolution by averaging (we increased the number of participants relative to the Experiment 1 to 24, since the counterbalancing rule required testing participants in groups of 8). Secondly, we increased the number of blocks, and thus of trials, used to estimate the PSS for simultaneity. Thirdly, we included a baseline phase at the beginning of the experiment.

During the first session of the experiment participants completed firstly a baseline phase and then 28 test blocks, each test block was composed of a learning phase followed by a short test phase. The second session of the experiment took place another day and consisted of 44 test blocks.

**Baseline phase.** At the beginning of the experiment participants completed a baseline phase. They were asked to perform left/right key-presses that triggered on a random basis one of the two possible audiovisual pairs. The timing of the auditory stimulus relative to the visual stimulus was randomly varied: -300, -233, -166, -100, -66, 0, 66, 100, 166, 233, and 300 ms. These 11 SOAs were presented 24, 28, 32, 36, 40, 40, 40, 36, 32, 28, 24 times respectively over the whole experiment; thus, each audiovisual pair was presented a total of 180 times. Participants’ were asked to judge whether the sound and the flash were presented simultaneously or not.

**Learning phase.** Each learning phase consisted of 20 trials except for the first block of each session in which the learning phase consisted of 100 trials (for a total of 1620 learning trials in the whole experiment). The rest was exactly the same as for the learning phases of our main experiment.

**Test phase.** In the test phase the timing of the auditory stimulus relative to the visual stimulus was randomly varied and selected from the following stimulus onset asynchrony: -300, -230, -166, -100, -66, 0, 66, 100, 166, 232, and 300 ms. These 11 SOAs were presented 48, 56, 64, 72, 80, 80, 80, 72, 64, 56, and 48 times respectively for a total of 720 trials. In more details, participants completed 180 trials per condition: i.e., 180 x 2 adaptation order x 2 Audiovisual pairs (predicted pairs and unpredicted pairs). The rest of the test phase was the same as the test phase of our main experiment.

**Results**

**Baseline.** We conducted two two-tailed paired t-tests on PSS and SD comparing trials in which participants performed a left key-press vs trials in which they executed a right key-press. As expected we did not find any PSS and SD differences when audiovisual pairs were generated with the left or the right hand (p = .383 and p = .298, respectively). Thus, for further analyses we pooled together left and right key-press trials. A single sample t-test showed that on average participants perceived audiovisual simultaneity when the sound was presented after the visual stimulus (PSS = 22 (± 41) ms) t(23) = 2.564, p = .017, d = 0.535.

**Test phase.** We conducted a repeated measure ANOVA on PSS with Adaptation order (sound first, sound second) and Audiovisual pair (predicted, unpredicted) as factors. We observed no main effect of Adaptation order F(1,23) = 2.346, p = .139, and no main effect of Audiovisual pair F(1,23) = .282, p = .600. However, the interaction Adaptation order * Audiovisual pair was significant F(1,23) = .5.155, p = .033,
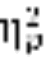
 = .183. A paired two-tailed t-test showed that PSS values shifted toward the adapted lag only when participants were presented with predicted audiovisual pairs, t(23) = -3.502, p = .002, *d* = 0.338. Notably, subjective audiovisual simultaneity for predicted pairs, when participants were adapted to audio before vision, was 21 ms (on average; positive values indicate sound after the flash). Instead, subjective audiovisual simultaneity for predicted audiovisual pairs, when participants were adapted to audio after vision, was 38 ms. Thus, the adaptation effect for predicted audiovisual outcomes, estimated by the difference between sound first and sound second adaptations, was 17 ms. Importantly, no change in PSS was observed for unpredicted trials (sound first average PSS = 33 ms, sound second average PSS = 31 ms; p = .841). Thus, we replicated the results of our main experiment showing that action-outcome learning drives audiovisual recalibration. As for the other three experiments, we observed a general bias in all conditions in perceiving audiovisual simultaneity when sounds were presented after the flash (sound first predicted pair: t(23) = 2.086, p = 0.048, d = 0.435; sound first unpredicted pair: t(23) = 2.632, p = 0.015, d = 0.549; sound second predicted pair t(23) = 3.675, p = 0.001, d = 0.766; sound second unpredicted pair t(23) = 3.128, p = 0.005, d = 0.652) . This might suggest that participants perceived in general sounds faster than flashes (see also Roseboom, Kawabe, & Nishida, 2013; van Eijk, Kohlrausch, Juola, & van de Par, 2008).

The same analyses on SD values showed no main effect of Adaptation, Audiovisual pair and no significant interaction F(1, 23) = 2.746, p = .111, F(1,23) = 2.767, p = .110, and F(1, 23) = 1.190, p = .287 respectively.

We then compared PSS values of the baseline phase with the values obtained in the test phase. Only the comparison between sound second predicted pair and baseline was significant t(23) = 2.750, p = .011, d = 0.360. The average PSS difference between test and baseline was as follow (PSS values in the baseline were subtracted from PSS values in the test phase): sound first predicted pair PSSdiff = -0.5 (±23) ms; sound first unpredicted pair PSSdiff = 11 (±33) ms; sound second predicted pair PSSdiff = 16 (±28) ms; sound second unpredicted pair PSSdiff = 10 (±31) ms. Overall, in the test phase there was a tendency in perceiving simultaneity when sounds were presented more strongly after the visual stimulus compared to the baseline.

The same analyses on SD values showed that on average temporal sensitivity decreased in the test compared to the baseline phase: the average SD in the test phase was 173 (± 64) ms vs 147 (±41) ms, p = .008.


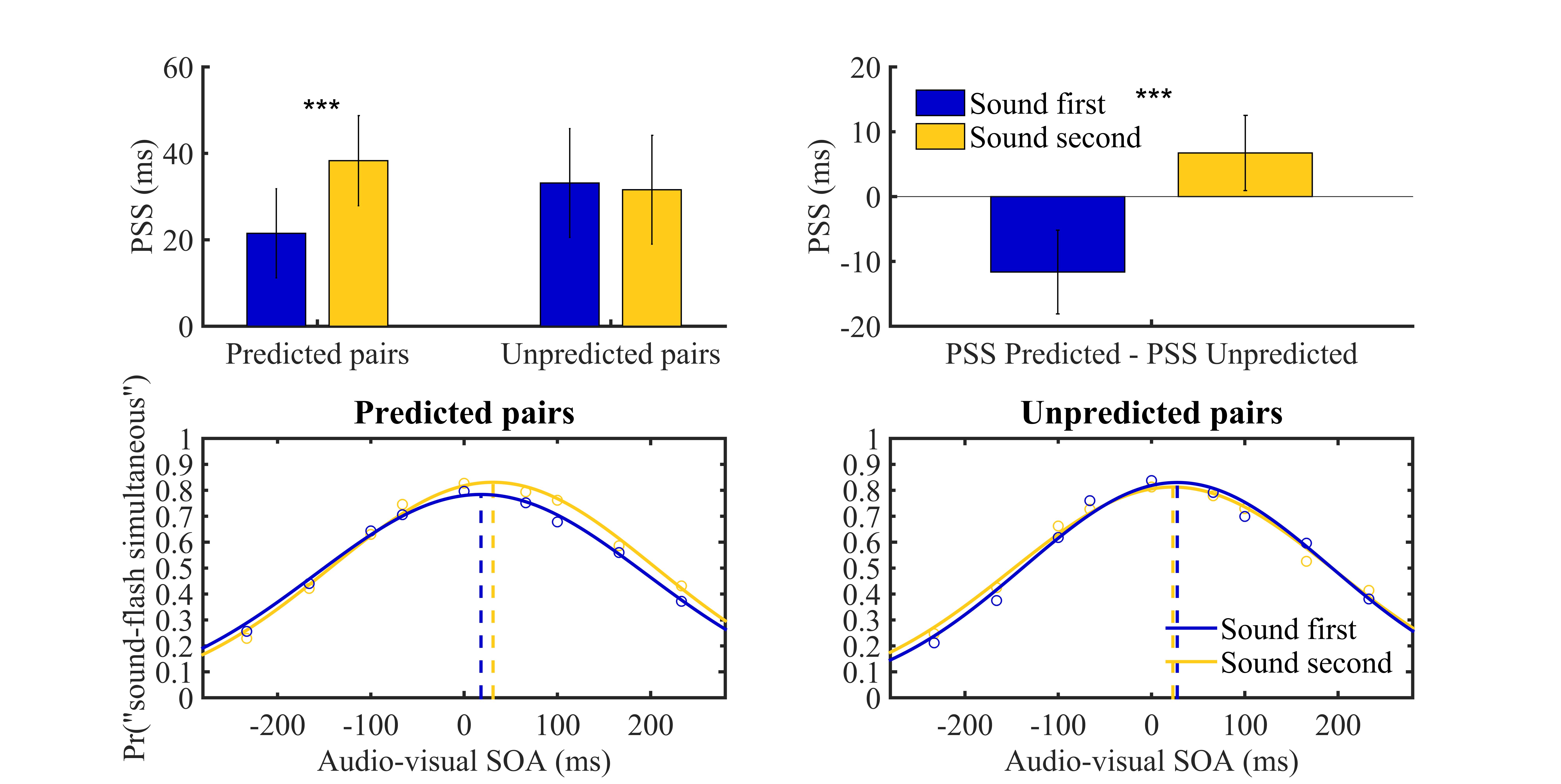


**Figure 4. (**Top left panel) Mean PSS values for both Adaptation order and Audiovisual pairs (averaged across all participants). Positive PSS indicates that participants perceived audiovisual simultaneity when sounds were presented after the flash. Bars represents standard errors. (Top right panel) We subtracted PSS values for unpredicted pairs from PSS value for predicted pairs both for sound first and sound second adaptation. (Bottom panels) Proportion of “sound and flash simultaneous” responses for predicted (left panel) and unpredicted pairs (right panel) for both adaptation orders (averaged across all participants) as a function of SOA.

**Discussion**

As in our main study we observed that audiovisual recalibration of simultaneity occurred only when actions triggered the presentation of predicted audiovisual pairs but not when participants were presented with unpredicted audiovisual pairs (i.e., the audiovisual pair presented after the action had been associated with the *other* action in the learning phase). This suggests that learning the relation between an action and a specific audiovisual outcome pair drives temporal binding of the audio and visual components *within* the outcome pair.

**References**

Dienes, Z. (2014). Using Bayes to get the most out of non-significant results. Quantitative Psychology and Measurement, 5, 781. <http://doi.org/10.3389/fpsyg.2014.00781>.

Fujisaki W., Shimojo S., Kashino M., Nishida S. (2004) Recalibration of audiovisual simultaneity. Nature Neuroscience, 7 (7) (2004), pp. 773–778 http://dx.doi.org/10.1038/nn1268

Roseboom, W., Kawabe, T. & Nishida, S. (2013). Audio-visual temporal recalibration can be constrained by content cues regardless of spatial overlap. Percept. Sci. 4, 189.

Eijk, R. L. J. van, Kohlrausch, A., Juola, J. F. & Par, S. van de. (2008) Audiovisual synchrony and temporal order judgments: Effects of experimental method and stimulus type. Percept. Psychophys. 70, 955–968.

1. Note that 8 more participants were initially recruited but not included in the sample size. 5 of them were not included in the sample because they showed a temporal discrimination (SD) higher than the longer SOA (300 ms) in the baseline phase. The other 3 participants did successfully complete the baseline phase but were excluded for other reasons. Two of them because they exhibited very low temporal discrimination in the test phase, the other because the amplitude of the psychometric curve did not reach a minimum of ~0.5 proportion of judgment simultaneous: the participant’s highest proportion of judgment sound-flash simultaneous was 0.23. [↑](#footnote-ref-2)
